# Compressive Properties and Constitutive Model of Semicrystalline Polyethylene

**DOI:** 10.3390/polym13172895

**Published:** 2021-08-27

**Authors:** Kebin Zhang, Wenbin Li, Yu Zheng, Wenjin Yao, Changfang Zhao

**Affiliations:** 1 ZNDY of Ministerial Key Laboratory, Nanjing University of Science and Technology, Nanjing 210094, China; kb2018@njust.edu.cn (K.Z.); zhengyu@njust.edu.cn (Y.Z.); njyaowj@163.com (W.Y.); 2 School of Mechanical Engineering, Nanjing University of Science and Technology, Nanjing 210094, China; lackychang@njust.edu.cn

**Keywords:** polyethylene, constitutive model, strain rate, temperature, finite element simulation

## Abstract

The mechanical properties of polyethylene (PE) materials are greatly influenced by their molecular structures, environmental temperature, and strain rate. In this study, static and dynamic compression tests were performed on two semicrystalline PE materials—ultrahigh molecular weight polyethylene (UHMWPE) and high-density polyethylene (HDPE). The stress–strain curves of HDPE and UHMWPE under uniaxial compression at temperatures of −40–120 °C and strain rates of 0.001–5500 s^−1^ were obtained. The research findings suggest that both the UHMWPE and HDPE showed significant strain rate-strengthening effect and temperature-softening effect. In particular, HDPE exhibited better compression resistance and high-temperature resistance. The relationships between the yield stress and temperature and between the yield stress and strain rate for both materials were fitted, and the Cowper–Symonds constitutive model was built while considering the temperature effect. The parameters of the constitutive model were obtained and input into LS-DYNA software to simulate the dynamic compression process of HDPE. The simulation result was consistent with the test result, validating the accuracy of the constitutive parameters.

## 1. Introduction

To improve the insensitivity of ammunition during design, multiple small pressure relief holes, in which low-melting-point materials are stuffed, are usually designed at the bottom or top of the projectile body [1]. The projectile will be heated by an external heat source such as fire. When the charge is heated to a certain temperature, the low-melting-point materials stuffed in the pressure relief holes will be softened and discharged to release the high-temperature and high-pressure gas generated by the decomposing charge and reduce the temperature and pressure within the projectile body; this can lower the reaction level of the ammunition, minimize accident-caused property loss, and ensure the staff safety [2,3,4]. Chen et al. [5] compared the reaction characteristics of four low-melting-point materials—polyethylene (PE), polybutylene terephthalate, polyamide 6, and polycarbonate—under the action of temperature, pressure, and temperature–pressure coupling in a self-made device. The results showed that PE could more easily create exhaust ducts at the charge reaction temperature than the other three materials.

Polyethylene is a high-quality material with favourable chemical stability, impact resistance, and low-temperature resistance. It has been widely used in various fields, such as firearm technology, transportation, medical instrumentation, biocomposites, construction, and retrofit [6,7,8]. Daniel et al. [9] used two different series of bio-based polyethylene (BioPE) to manufacture biocomposites, supplemented by thermomechanical pulp (TMP) fibers, and the results showed that BioPE’s 3D printing has been improved by adding 10% and 20% of TMP to the composition fiber. Rousakis et al. [10] found that using UHMWPE tape, aramid fiber tape, or basalt fiber tape to wrap the cylinder can significantly enhance the mechanical properties of concrete under monotonic or cyclic loading. Li et al. [11] used a polymer solution crystallization method to periodically modify UHMWPE fibers with polymer layered crystals to form a bamboo-like structure, and found that bamboo-like UHMWPE fiber-reinforced epoxy resin (EP) composites have higher strength and toughness. Singh et al. [12] found that adding a certain amount of rice husk fiber to HDPE can significantly improve the tensile strength and flexural strength of HDPE. Sphsa et al. [13] studied the load transfer mechanism in the bolted joints of laminates made of UHMWPE through experiments and analytical models, and obtained the load transfer properties and failure mechanism of laminates by using the joint geometry and the transverse clamping force on the laminate surface. Marcelo et al. [14] made polycaprolactone (PCL) and UHMWPE sheets through a single point incremental molding process (SPIF), and conducted in-depth characterization of the thermal and structural properties of the two polymers.

The melting point of polyethylene is between 110 °C and 140 °C, which is particularly suitable for the design and manufacture of low melting point parts for insensitive ammunition [5]. Besides the melting point of materials, the influence of the projectile overload and temperature on insensitive ammunition parts should also be considered during design. Some studies indicate that the air temperature range of the projectile at work is −40–50 °C [15], the maximum storage temperature is approximately 70 °C, and the strain rate can reach 10^3^ s^−1^ [16]. Thus, it is necessary to study the dynamic and static mechanical performances and the constitutive model of PE at different temperatures.

Several studies have been performed on the constitutive models of PE materials. Van Dommelen et al. [17] established a new semicrystalline polymer constitutive model by introducing the Eyring flow rule on the basis of the micromechanical constitutive model, and the new model well predicted the uniaxial compression mechanical behaviour of HDPE under quasistatic conditions. Drozdov et al. [18] derived and established the constitutive equation of the nonlinear viscoelastic behaviour of amorphous glassy polymers in the subyield zone, which was verified by mechanical tests and numerical simulations of polycarbonate. Bergström et al. [19] compared the effectiveness of the “J2-plasticity” theory [20], the “Arruda–Boyce” model [21,22], the “Hasan–Boyce” model [23], and the “Bergström–Boyce” model [24] to reproduce the tensile and compressive mechanical behaviours of UHMWPE under quasistatic conditions, and they also developed a hybrid model that could effectively predict the tensile and compressive mechanical behaviours under quasistatic conditions by combining previous theories. However, they failed to consider the influence of strain rate on the UHMWPE mechanical properties. Xu et al. [25] investigated the mechanical properties of PE at strain rates of 0.001–3204 s^−1^. The mechanical behaviours of PE under uniaxial compression in the elastic stage, yield stage, and plastic stage were described by constructing segmented constitutive models. However, the influence of temperature on the PE mechanical properties was not considered. Zhang et al. [26] studied the deformation behaviour of UHMWPE at strain rates of 0.001–3300 s^−1^ and temperatures of −40–100 °C. A constitutive model of UHMWPE in the plastic stage under dynamic conditions was built, but it could not describe the mechanical behaviour of UHMWPE in the elastic stage and could hardly be used in finite element simulation. To this end, the current study aimed to build a strain rate–temperature constitutive model of PE materials that can be used for finite element simulation, so as to provide reference for the design of pressure relief components of insensitive ammunition.

Both HDPE and UHMWPE are semicrystalline thermoplastic polymers with higher strength than all other PE materials; thus, they can function as materials for the pressure-relief parts of insensitive ammunition. To compare the compressive mechanical performances of HDPE and UHMWPE and obtain their constitutive models, both materials were subjected to static and dynamic compressive performance tests in this study. Based on the test results, the stress–strain curves of HDPE under uniaxial compression at temperatures of −40–120 °C and strain rates of 0.001–5450 s^−1^ were obtained. Further experimental investigation was conducted based on the study by Zhang et al. [26] to obtain the stress–strain curve under uniaxial compression at temperatures of −40–100 °C and strain rates of 0.001–5500 s^−1^. In addition, an FEI Quanta 250 F field-emission environmental scanning electron microscope was used to observe and analyze the microscopic morphology of UHMWPE and HDPE. The yield stresses of HDPE and UHMWPE at different strain rates and temperatures were fitted, and the Cowper–Symonds constitutive model that included the temperature effect was established. The Cowper–Symonds constitutive model was applied to the 89# material model [27] in LS-DYNA to simulate the dynamic compression of HDPE. The results validated the accuracy of the constitutive model.

## 2. Materials

### 2.1. Sample Preparation

Polyethylene is a thermoplastic resin prepared via vinyl polymerization. High-density PE and UHMWPE are two typical PE materials. The former is polymerized at low pressures and has higher crystallinity and density than common PE materials. Compared with HDPE, UHMWPE has higher molecular weight and longer chain segments.

The HDPE plates used in this study were produced using a plastic extruder. To prepare the material, HDPE powder at 220 °C was delivered to the feed port of the extruder where the speed was 75 rpm. The extruded mixture was pressed into an extrusion roller (diameter: 320 mm, width: 1000 mm). The material was then cooled to about 25 °C by circulating water before it was cut into square plates of 20 mm × 400 mm × 400 mm. By contrast, the UHMWPE plates were obtained via compression moulding following the ISO9001 standards. The raw UHMWPE material purchased from Ticona GmbH (Hearst, Germany) was poured into a high-speed mixer. After mixing, the mixture was placed into a hot press for hot pressing for 2–8 h and then subjected to cold pressing in a cold press for another 2–8 h. Afterwards, the blank was fed to a pressure-keeping machine for cooling and shaping before it was prepared into UHMWPE plates of 1000 mm × 1000 mm × 15 mm [26]. The material properties provided by the manufacturers are listed in Table 1.

Specimens of both materials were prepared on a lathe. The specimens for static compression were cylinders with 10 mm height and 10 mm diameter. Two different types of dynamic-compression specimens (ϕ10×5 mm and ϕ7×3.5 mm) were used in this study. With a length/diameter ratio of 0.5, the influence of the inertia effect and the end-friction of specimens could be effectively reduced [28]. As shown in Equation (7), a higher strain rate could be obtained by reducing the specimen thickness. To ensure material consistency, the axes of all the specimens were perpendicular to the plate surface.

**Figure 1:**
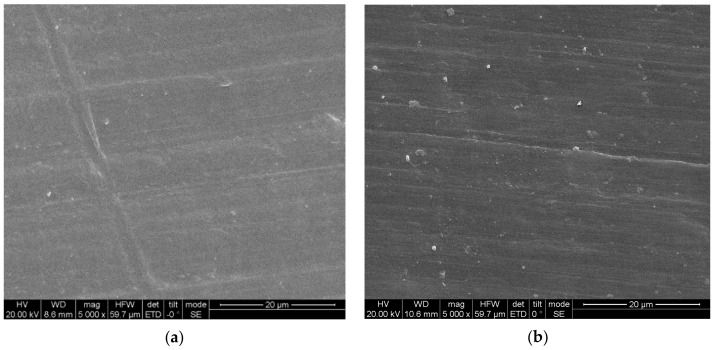
Scanning electron microscopy images of (**a**) HDPE and (**b**) UHMWPE.

After the specimens were cut along the axial direction and sprayed, the microscopic morphologies of HDPE and UHMWPE were observed using the FEI Quanta 250 F field-emission environmental scanning electron microscope. As shown in Figure 1, HDPE and UHMWPE showed similar microscopic morphologies. Lamellas grew epitaxially, with the fibre direction the same as the direction of the molecular chain axis in both materials. Both materials featured a shish-kebab structure and large diameters [29]. Owing to the pressure effect on the fibre crystals, the crystals grew with different diameters. There was a large distortion in the direction of the coarse fibres, which made some fibre crystals interpenetrate and intertwine in the growth direction. This was more noticeable in the scanning electron microscopy (SEM) image of HDPE. Numerous small particles of approximately 1 μm diameter occurred in the crystal structures of both materials, with more small particles occurring in UHMWPE than in HDPE.

### 2.2. Differential Scanning Calorimetry Test

**Figure 2:**
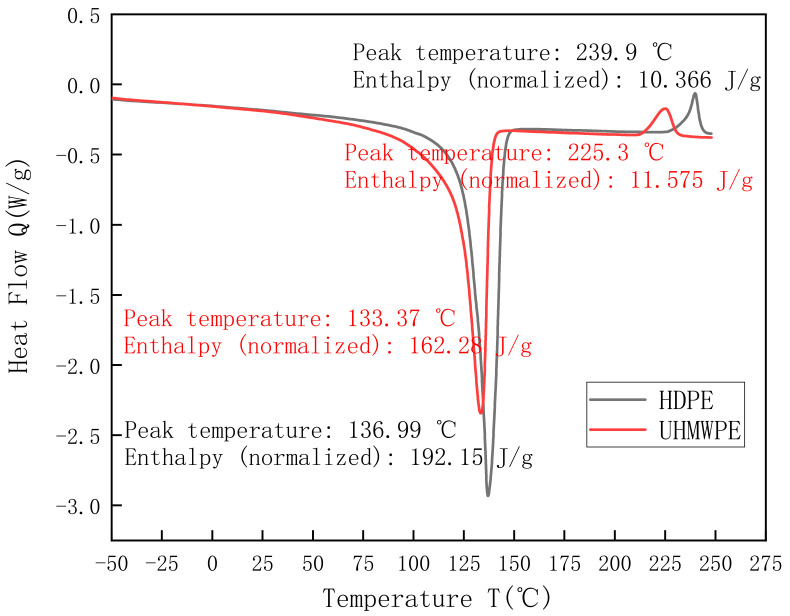
Differential scanning calorimetry curves of HDPE and UHMWPE.

To obtain the crystallinities and melting points of HDPE and UHMWPE, a differential scanning calorimeter was used to perform thermal analysis tests. During the test, the masses of HDPE and UHMWPE samples were 7.8 mg and 7.3 mg, respectively. The sample temperature increased from −50 °C to 300 °C at a rate of 10 K/min. The differential scanning calorimetry (DSC) curves of HDPE and UHMWPE are displayed in Figure 2.

As shown in Figure 2, the DSC curves of HDPE and UHMWPE differed significantly because of the difference in the lamella thickness of PE [30]. Moreover, HDPE featured a higher melting point and decomposition temperature than UHMWPE. The crystallinities of HDPE and UHMWPE were obtained as 71.2% and 56.3%, respectively, using Equation (1) [31]. This indicates that although there were few branched chains in both materials, the crystallinity of HDPE was higher because the too-large molecular weight of UHMWPE impedes crystallization.

(1) $$\mathrm{Crystallinity}=\frac{\Delta H_{f}}{\Delta H_{m}}\times 100\%$$

where $\Delta H_{f}$ is the enthalpy of fusion per unit mass of the sample, and $\Delta H_{m}$ is the heat of fusion of the sample at 100% crystallization (Table 1).

## 3. Test Method

### 3.1. Quasi-Static Testing

To identify the influence of temperature and strain rate on the HDPE and UHMWPE mechanical properties at a low strain rate, the specimens of both materials were subjected to a uniaxial compression test using an MTS tester at three speed levels: 0.6 mm/min, 6 mm/min, and 60 mm/min. Moreover, the compression test was performed at different specimen temperatures: −40 °C, −20 °C, 25 °C, 50 °C, 70 °C, 90 °C, and 120 °C, using an air compressor and an electrothermal furnace. The specimen temperature was preserved for 5 min before each test to ensure that it was the same as the test temperature [32]. To ensure the test data accuracy, an extensometer was used to record specimen deformation during the test. Lubricating grease was applied to the end surfaces of the specimens and tools to reduce friction during the test. For error reduction, the test was repeated three times for each working condition. Therefore, three sets of test data on loading force *F* (kN) and displacement *S* (mm) were collected, and the averages were taken for data processing.

According to the definitions of stress and strain, the *F*–*S* curves collected were converted into engineering stress $\sigma_{E}$ and engineering strain $\varepsilon_{E}$:

(2) $$\sigma_{E}=\frac{F}{A_{s}},$$

(3) $$\varepsilon_{E}=\frac{L_{s}-L_{i}}{L_{s}},\;$$

(4) $$S=L_{s}-L_{i},$$

where *F* is the loading force, *S* is the displacement, $A_{s}$ and $L_{s}$ are the initial cross-sectional area and length of the specimen, respectively, and $L_{i}$ is the instantaneous length of the specimen.

Furthermore, the engineering strain and stress of materials were converted into true stress–strain curves using Equations (5) and (6), respectively.

(5) $$\sigma_{T}=\sigma_{E}\left(1-\varepsilon_{E}\right),\;$$

(6) $$\varepsilon_{T}=-\ln\left(1-\varepsilon_{E}\right),\;$$

### 3.2. Dynamic Testing

**Figure 3:**
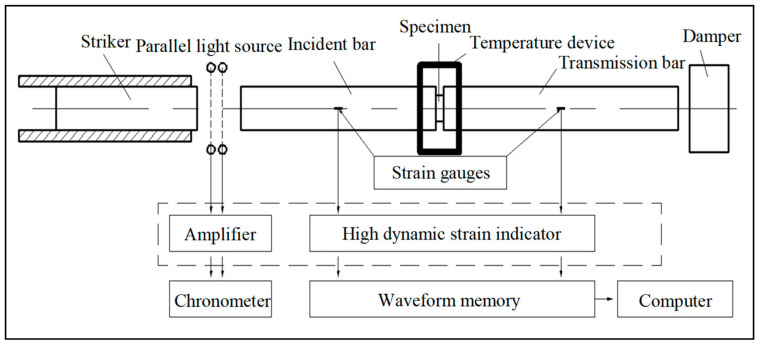
The split Hopkinson pressure bar testing device.

The split Hopkinson pressure bar (SHPB) technique is a major approach for testing the stress–strain relationships of materials at high strain rates of 10^2^–10^4^ s^−1^ [33]. Figure 3 displays a diagram of the SHPB test device used in this study. Considering that both HDPE and UHMWPE are low-impedance materials, 7A04 aluminium alloy bars were used in the test to ensure a high signal-to-noise ratio of the data obtained [34]. Detailed parameters of the SHPB used are listed in Table 2.

To investigate the dynamic compressive mechanical properties of the two materials at different temperatures, a liquid nitrogen furnace and a heating furnace were used to cool and heat the specimens, respectively. The HDPE and UHMWPE specimens were subjected to dynamic compressive mechanical property test at temperatures of −40 °C, −20 °C, 25 °C, 50 °C, 70 °C, 90 °C, and 120 °C. The temperatures of the specimens and the bar sections contacting the specimens were preserved for 5 min before each test to ensure that the specimen temperature and test temperature were consistent [8]. In the test, an empty bar test was carried out at various temperatures. According to the test results, the bar performance was not affected by temperature. Moreover, the test was repeated three times at each temperature and strain rate, and the results were averaged to ensure data accuracy.

Based on the strain signals $\varepsilon_{r}\left(t\right)$ and $\varepsilon_{t}\left(t\right)$ collected by the strain gages fixed on the incident and transmission bars, the $\sigma_{E}$, $\varepsilon_{E}$, and nominal strain rate $\dot{\varepsilon }$ [35,36] in the strain process were calculated using the following equations:

(7) $$\dot{\varepsilon }=-\frac{2C_{0}}{L_{S}}\varepsilon_{r}\left(t\right),$$

(8) $$\varepsilon_{E}=\int_{0}^{t}\dot{\varepsilon }dt,$$

(9) $$\sigma_{E}=\frac{A_{0}E_{0}}{A_{S}}\varepsilon_{t}\left(t\right),$$

where $A_{S}\;$ is the initial cross-sectional area of the specimen, $L_{S}$ is the initial length of the specimen, *C*_0_ is the elastic wave velocity of the bar, *A*_0_ is the cross-sectional area of the bar, and *E*_0_ is the elastic modulus of the bar. The true stress $\sigma_{T}$ and strain $\varepsilon_{T}$ of the specimen could be obtained using Equations (5) and (6), respectively.

## 4. Results and Discussion

### 4.1. Quasi-Static Test

**Figure 4:**
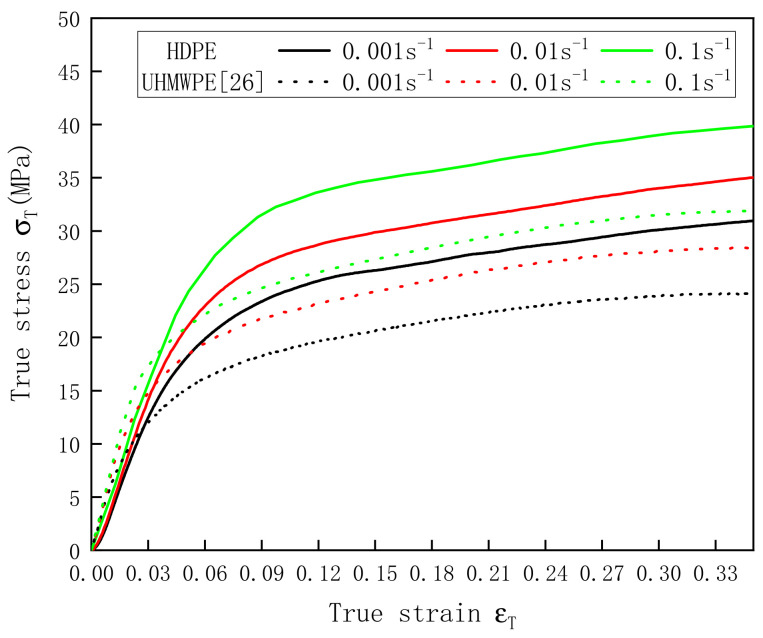
Stress–strain curves of HDPE and UHMWPE at strain rates of 0.001–0.1 s^−1^.

The stress–strain curves of HDPE and UHMWPE at 0.001–0.1 s^−1^ were obtained by processing the data collected using the MTS machine. The data acquired by Zhang et al. [26] are also included in Figure 4. Both PE materials showed strong viscoelastic plasticity. During compression, the materials first experienced a linear elastic stage, followed by a stepwise yield stage and then a plastic stage. Figure 4 also shows that the strain rate had a pronounced effect on the yield stresses and elastic moduli of HDPE and UHMWPE. With an increase in the strain rate, the yield stress and elastic modulus increased. However, the slopes of the two PE materials in the strain hardening section did not increase significantly with the strain rate.

**Figure 5:**
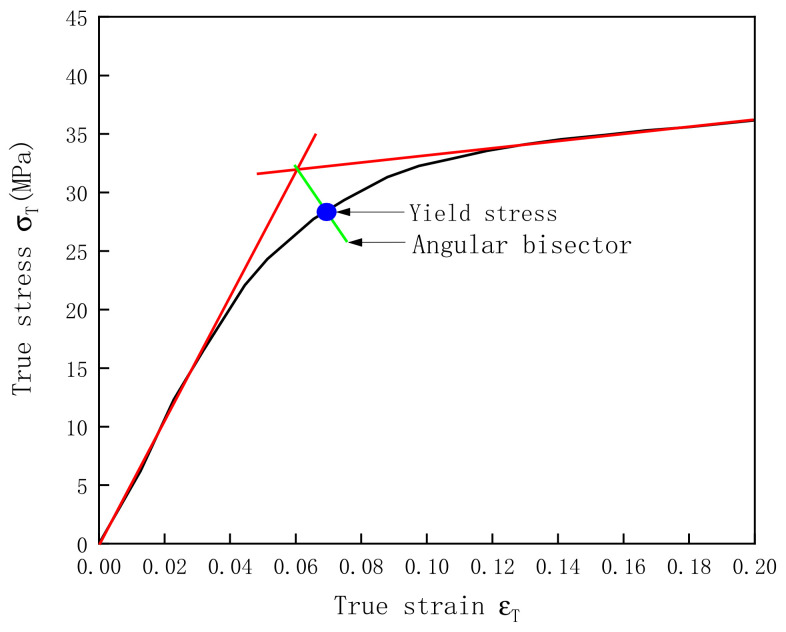
Method to obtain the yield stress of materials.

To more clearly compare the mechanical properties of HDPE and UHMWPE, their yield stresses are summarized in Table 3. Because it is difficult to determine the yield stress of PE materials, the stress obtained in Figure 5 was taken as the yield stress of the materials. From the data in Table 3, HDPE exhibited better compressive mechanical properties than UHMWPE.

**Figure 6:**
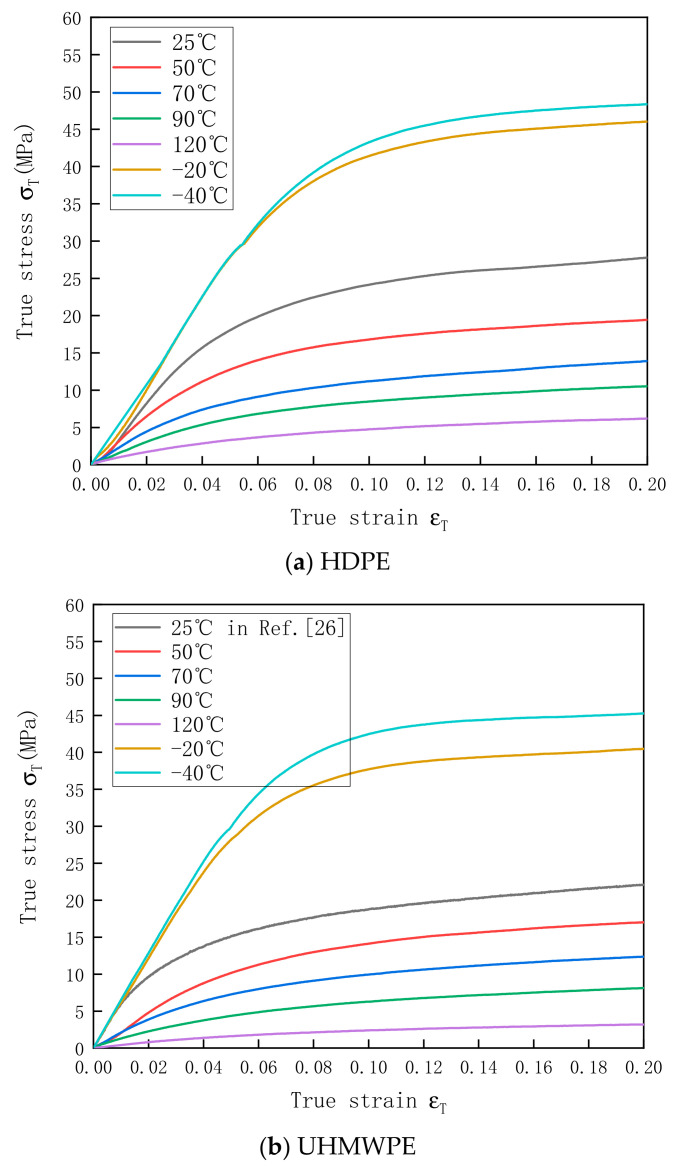
Stress–strain curves of (**a**) HDPE and (**b**) UHMWPE at a strain rate of 0.001 s^−1^ and temperatures of −40–120 °C.

The stress–strain curves of HDPE and UHMWPE at the strain rate of 0.001 s^−1^ and temperature range of −40–120 °C are shown in Figure 6a,b, respectively. The test results indicate that both HDPE and UHMWPE showed significant temperature-softening effect. With the increase in temperature, the elastic moduli of the materials decreased. In addition, the elastic stage and plastic stage of the materials gradually overlapped. Moreover, UHMWPE was almost hyper-elastic at 120 °C, suggesting that the heat deflection temperature of the material was reached and that the compressive resistance of the material was significantly reduced. According to the data in Table 3, HDPE exhibited better high-temperature resistance than UHMWPE.

### 4.2. Dynamic Test

**Figure 7:**
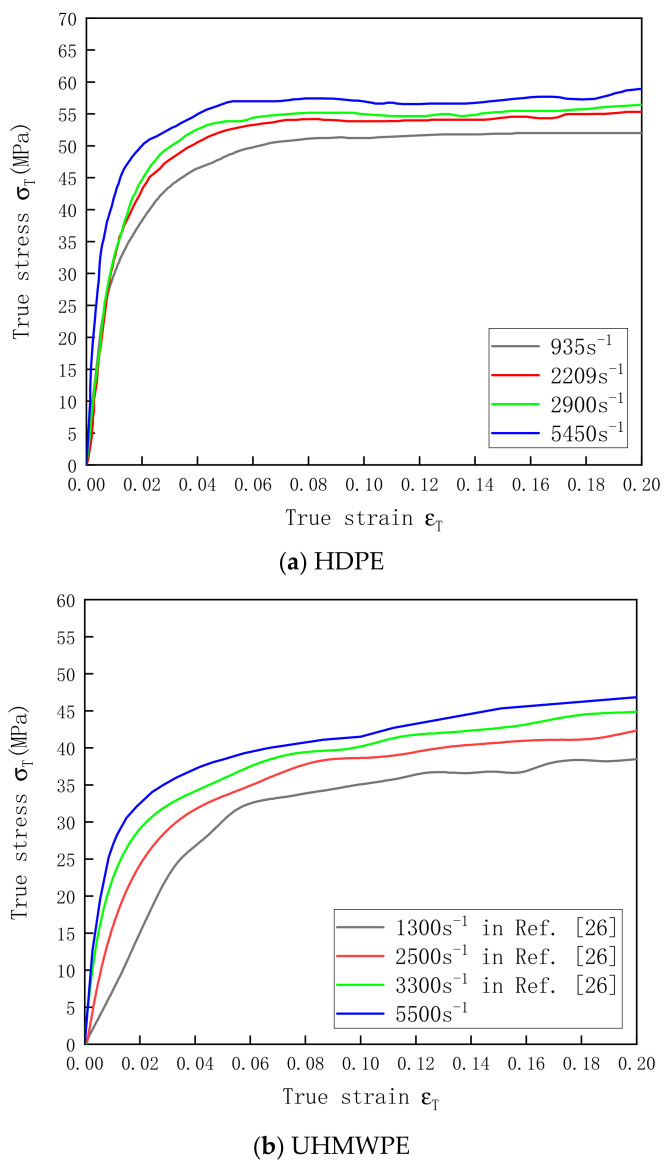
Stress–strain curves of (**a**) HDPE and (**b**) UHMWPE at a temperature of 25 °C under dynamic conditions.

The stress–strain curve of HDPE at strain rates of 935–5450 s^−1^ and that of UHMWPE at strain rates of 1300–5500 s^−1^ were obtained through the SHPB test. The data acquired by Zhang et al. [26] are also included in Figure 7b. The test results show that HDPE has better compressive mechanical properties than UHMWPE, and the two materials also exhibit strain rate effects under dynamic conditions. The yield stresses and the elastic moduli of the materials increased with the strain rate. Many researchers argue that this phenomenon is associated with the secondary molecular process of polymers [37,38]. Polyethylene chains can harden with the increase in strain rate, which will reduce the molecular mobility of chain segments and increase the yield stress [26].

**Figure 8:**
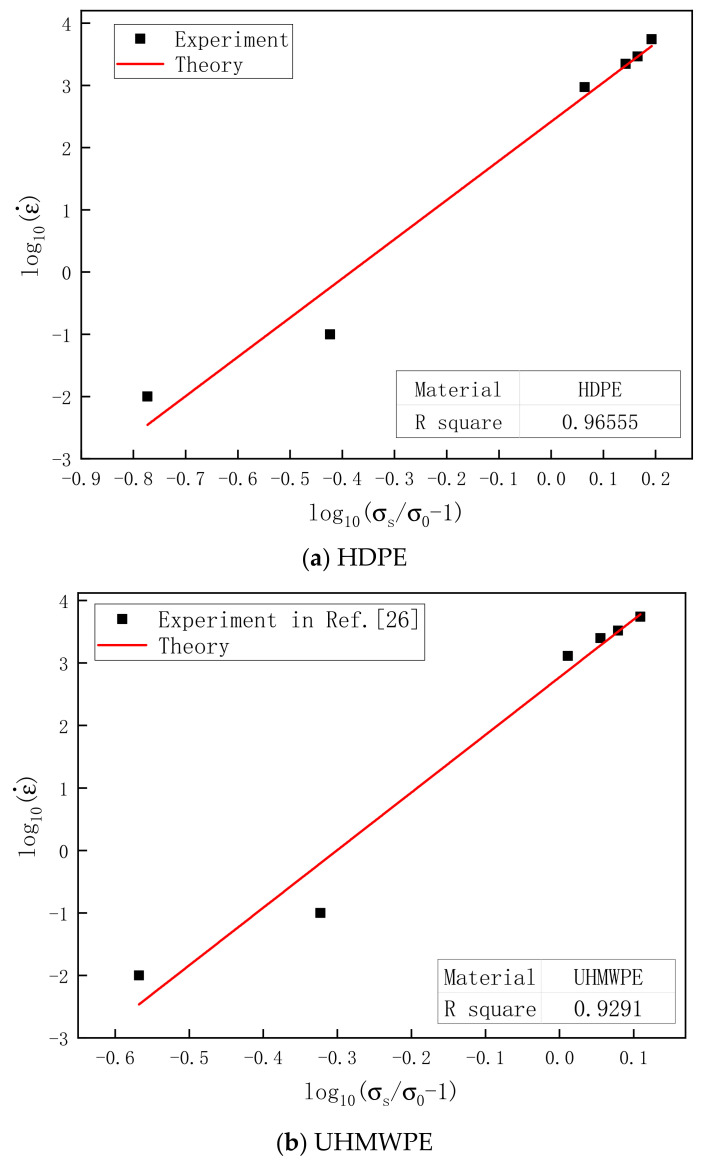
Relationship between the yield stress and strain rate for (**a**) HDPE and (**b**) UHMWPE.

Figure 8a,b shows the logarithmic relationship between the yield stress and strain rate of HDPE and UHMWPE at 25 °C. It can be found that the yield stress of the two materials has a linear logarithmic relationship with the strain rate.

**Figure 9:**
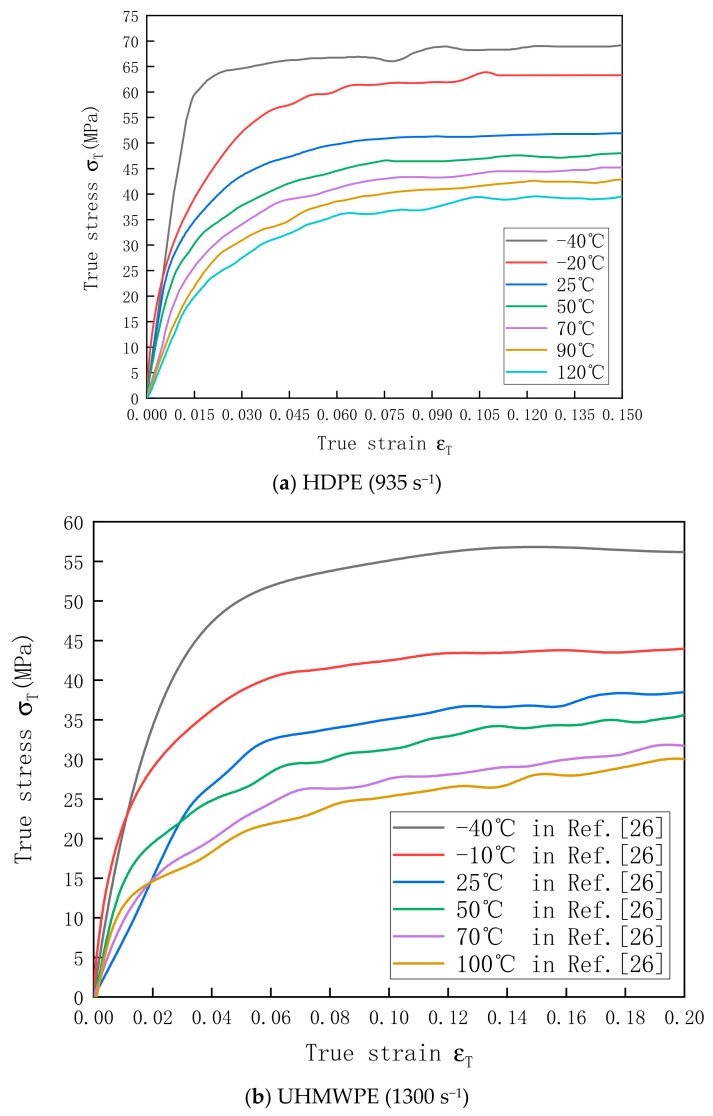
Stress–strain curves of (**a**) HDPE and (**b**) UHMWPE at different temperatures.

The stress–strain curves of HDPE (at an average strain rate of 935 s^−1^) and UHMWPE (at an average strain rate of 1300 s^−1^) at different temperatures are displayed in Figure 9a,b, respectively. According to the test results, HDPE and UHMWPE still showed strong temperature-softening effect at high strain rates.

**Figure 10:**
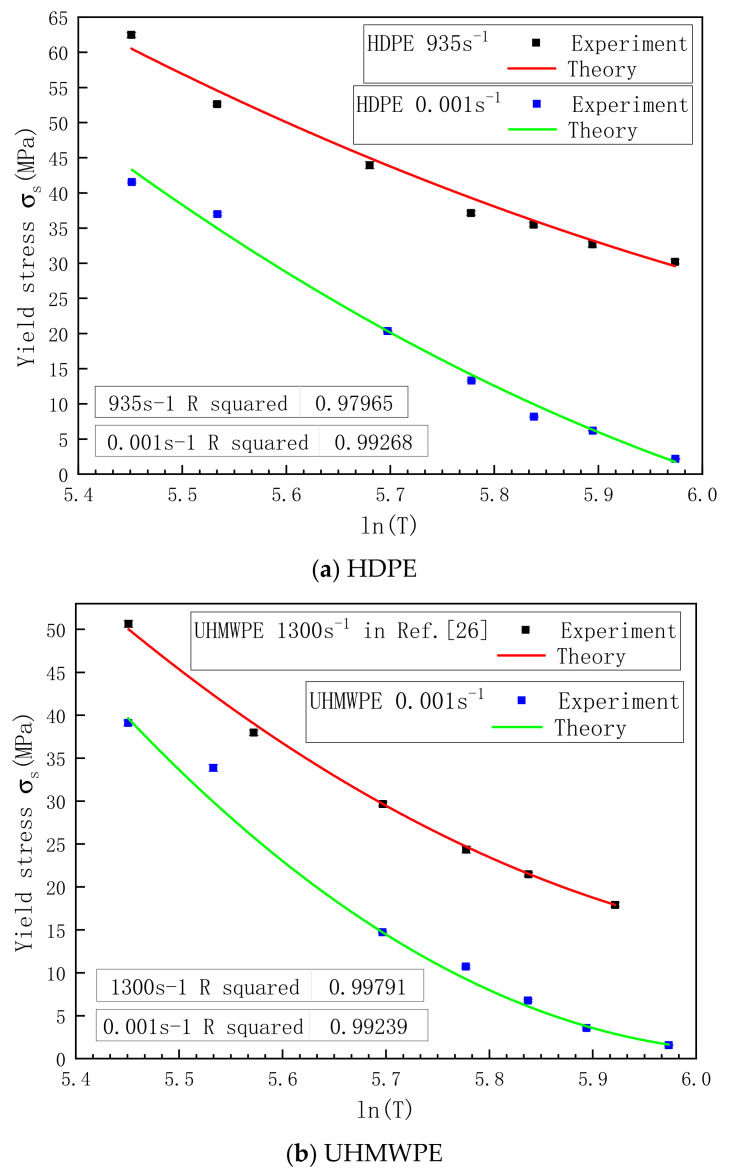
Relationship between the yield stress and temperature for (**a**) HDPE and (**b**) UHMWPE.

The relationships between the yield stress and temperature for HDPE and UHMWPE are illustrated in Figure 10a,b, respectively. The results indicate that the yield stress was nonlinearly correlated with temperature for both materials, and the nonlinear relationship was more prominent in UHMWPE. According to the comparison between Figure 10a,b, HDPE showed better high-temperature resistance than UHMWPE under the same conditions. From Figure 10, it can also be inferred that for both materials, the relationship between the yield stress and temperature under quasistatic conditions was different from that under dynamic conditions.

### 4.3. Constitutive Model

To facilitate the engineering calculations and applications, we did not consider the finite strain kinematics framework, but established the phenomenological constitutive model of HDPE and UHMWPE based on the experimental data and literature [27].

#### 4.3.1. Strain Rate Effect

The constitutive model of 89# material in LS-DYNA applies to materials whose stress–strain response and whose plastic part cannot be as clearly distinguished as in metals [27]. The simulation was performed by inputting the yield stress–strain rate relations and corresponding true stress–strain curves. Equation (10) is the relationship between yield stress and strain rate in the 89# material constitutive model, which is also known as the Cowper–Symonds model.

(10) $$\sigma_{s}=\sigma_{0}\left(1+{\left(\frac{\dot{\varepsilon }}{C}\right)}^{1/P}\right)$$

Take the logarithm of both sides of Equation (10) and simplify it to get:

(11) $$\log_{10}\dot{\varepsilon }=\log_{10}C+P\;\log_{10}\left(\frac{\sigma_{s}}{\sigma_{0}}-1\right)$$

where $\sigma_{s}$ is the yield stress of the material, $\sigma_{0}$ is the quasistatic (0.001 s^−1^) yield stress of the material, $\dot{\varepsilon }$ is the strain rate of the material, and P and C are material constants determined by test. According to the above findings, both HDPE and UHMWPE showed high viscoelastic plasticity and strong strain rate–strengthening effect. Hence, the relationships between the yield stress of both materials and strain rate were fitted based on Equation (11) (Figure 8). The values of parameters P and C obtained by fitting are listed in Table 4. The R^2^ of HDPE was 0.96555, and that of UHMWPE was 0.9291, indicating that Equation (10) was suitable for HDPE and UHMWPE in this study.

#### 4.3.2. Temperature Effect

According to the above findings, the yield stresses of HDPE and UHMWPE were nonlinearly correlated with temperature. The relationships between the yield stress and temperature of the two materials were different in quasistatic and dynamic conditions. Thus, the relationships between the yield stress and temperature of both materials under quasistatic and dynamic conditions were separately investigated. The relationship between the yield stress and temperature is expressed as follows, according to Zhang et al. [26]:

(12) $$\sigma_{s}=\sigma_{l}\left(1+FlnT_{*}+GlnT_{*}{}^{2}\right)$$

where $\sigma_{l}$ is the yield stress at the minimum strain rate during test (the quasistatic and dynamic strain rates of HDPE were 0.001 s^−1^ and 935 s^−1^, respectively; the quasistatic and dynamic strain rates of UHMWPE were 0.001 s^−1^ and 1300 s^−1^, respectively); $T^{*}=T/T_{r}$ is the dimensionless temperature; T is the test temperature; F and G are material constants determined by test; and $T_{r}$ is the reference temperature, which was 298 K in this study. All the temperatures were represented by thermodynamic temperature K during calculation. The fitting results are shown in Figure 10; parameters F and G obtained by fitting are listed in Table 4. According to the results, the R^2^ values of both materials fitted under quasistatic and dynamic conditions were larger than 0.97, which implies that for both HDPE and UHMWPE, the relationships between the yield stress and temperature can be well described by Equation (12).

### 4.4. Constitutive Model and Validation

Based on the consideration of the influence of temperature and strain rate effect on the yield stress of materials, the relationship between the yield stress, temperature, and strain rate for HDPE and UHMWPE is presented in Equation (13).

(13) $$\sigma_{s}=\sigma_{0}\left(1+{\left(\frac{\dot{\varepsilon }}{C}\right)}^{1/P}\right)\left(1+FlnT_{*}+GlnT_{*}{}^{2}\right)$$

**Figure 11:**
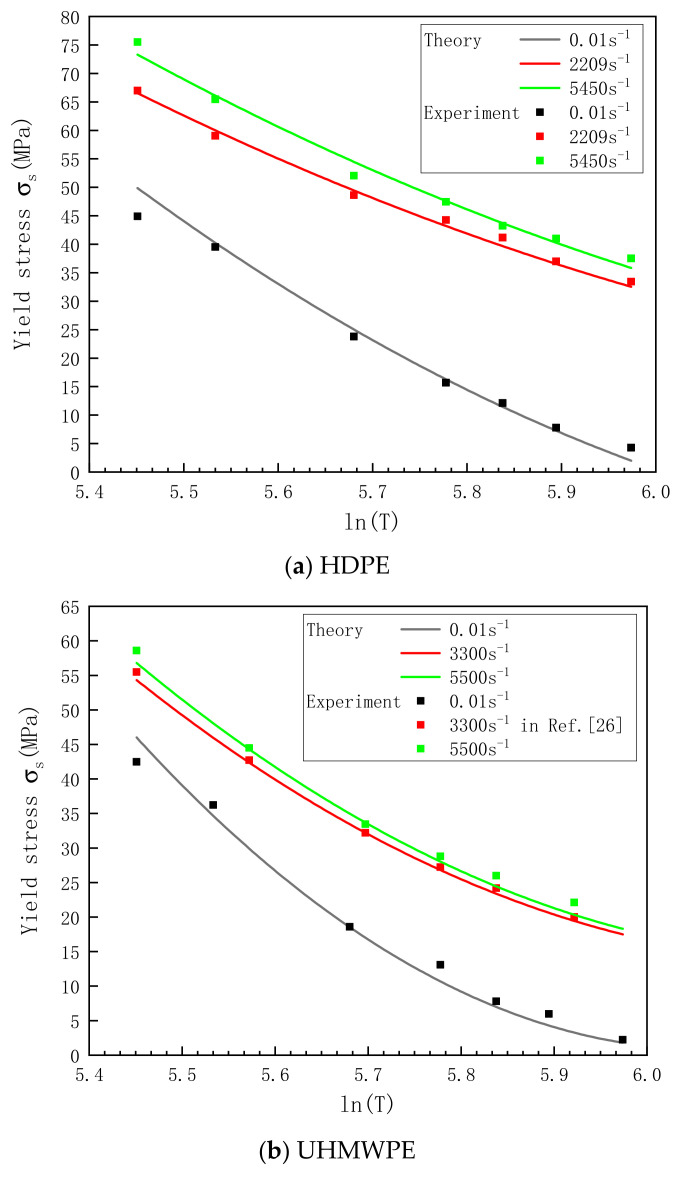
Relationship between the yield stress and temperature for (**a**) HDPE and (**b**) UHMWPE at different strain rates.

The parameters listed in Table 4 are substituted into Equation (13) to calculate the yield stresses of HDPE and UHMWPE at other temperatures and strain rates. As shown in Figure 11, the calculation result was compared with the test result. The comparison of the test results and the theoretical calculation values shows that the relationships between the yield stress of HDPE and UHMWPE and temperature at different strain rates can be well described by Equation (13).

However, the constitutive model established in this study still has certain limitations. After the large deformation of polyethylene occurs, the phenomenon of strain strengthening will appear (the stress increases sharply with the increase in strain) [39]. Our constitutive model cannot predict this strain strengthening phenomenon. Additionally, we only considered the temperature effect and did not introduce the thermal formula; therefore, the thermal-mechanical coupling (adiabatic and self-heating response) was not well captured.

### 4.5. Simulation

**Figure 12:**
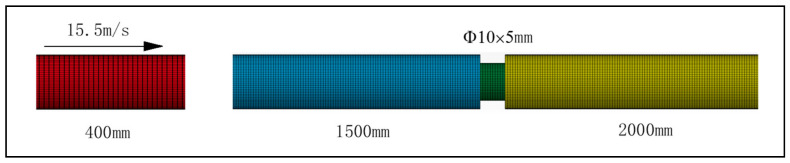
Finite element model of split Hopkinson pressure bar.

To further validate the accuracy of the proposed constitutive model, the HDPE constitutive model built in this study was inputted into LS-DYNA for simulation. An SHPB finite element model was built on a 1:1 scale. The element types of the models are all solid164 element. The Automatic Surface to Surface contact types were used between bars and bars, and between bars and specimens. The bullet impacted the incident bar at a velocity of 15.5 m/s (Figure 12). The SHPB parameters are listed in Table 5. The material model for HDPE specimens was the 89# material model in LS-DYNA, and the parameters are listed in Table 1; Table 4. During simulation, the true stress–strain curves of HDPE at different strain rates needed to be imported into the 89# material model. Considering that the SHPB test was dynamic, the stress–strain curve of HDPE at strain rates of 935–5450 s^−1^ was imported into the material model to improve the simulation result accuracy.

**Figure 13:**
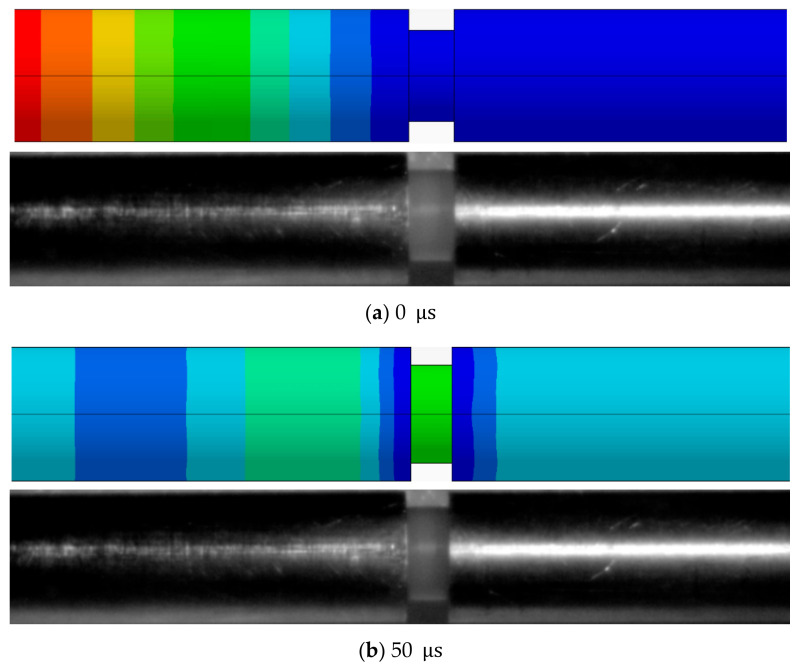

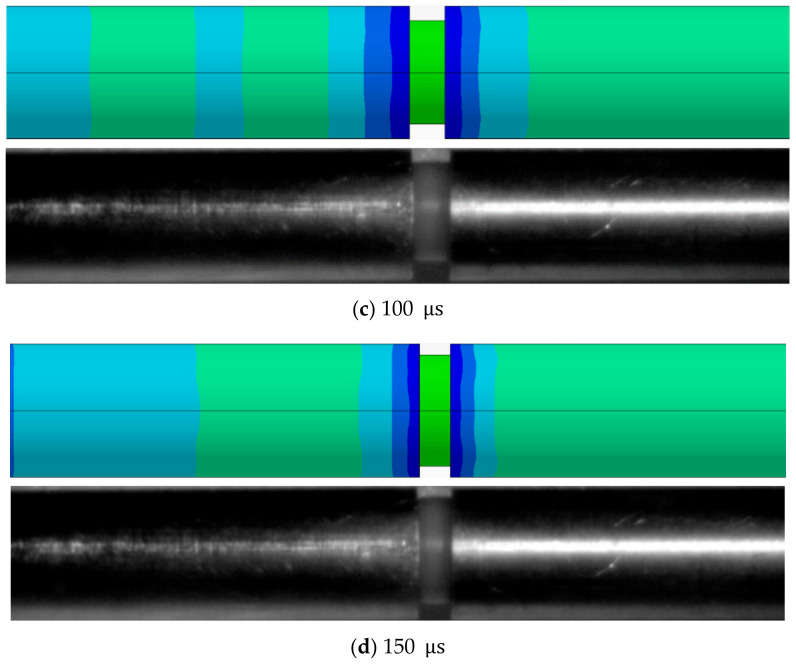
Comparison between specimen deformations in test and simulation at different times.

**Figure 14:**
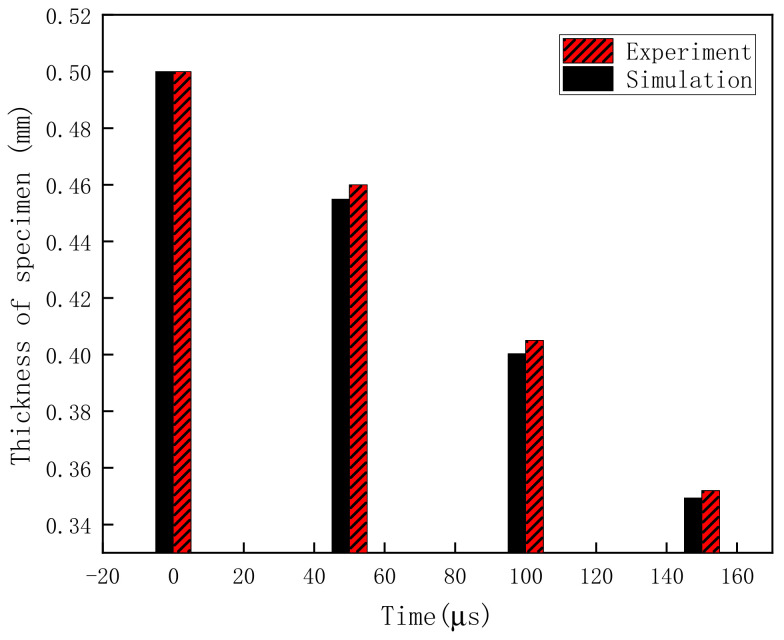
Comparison between specimen thicknesses in test and simulation.

To better explain the simulation accuracy, the deformation of HDPE specimens during the SHPB test was recorded using a high-speed camera and compared with the simulation result. Pictures were taken at 50 μs intervals. The time immediately before the specimen deformation was defined as the initial moment. The results of the specimen deformation from 0 μs to 150 μs obtained by the test and simulation are shown in Figure 13. According to the simulation result, the stress wave transmitted from the incident bar to the transmission bar through the HDPE specimen, which caused the specimen deformation. Figure 13; Figure 14 show that the variations in specimen thickness in the test and simulation were basically the same. In other words, the specimen deformation could be suitably predicted by the simulation model.

**Figure 15:**
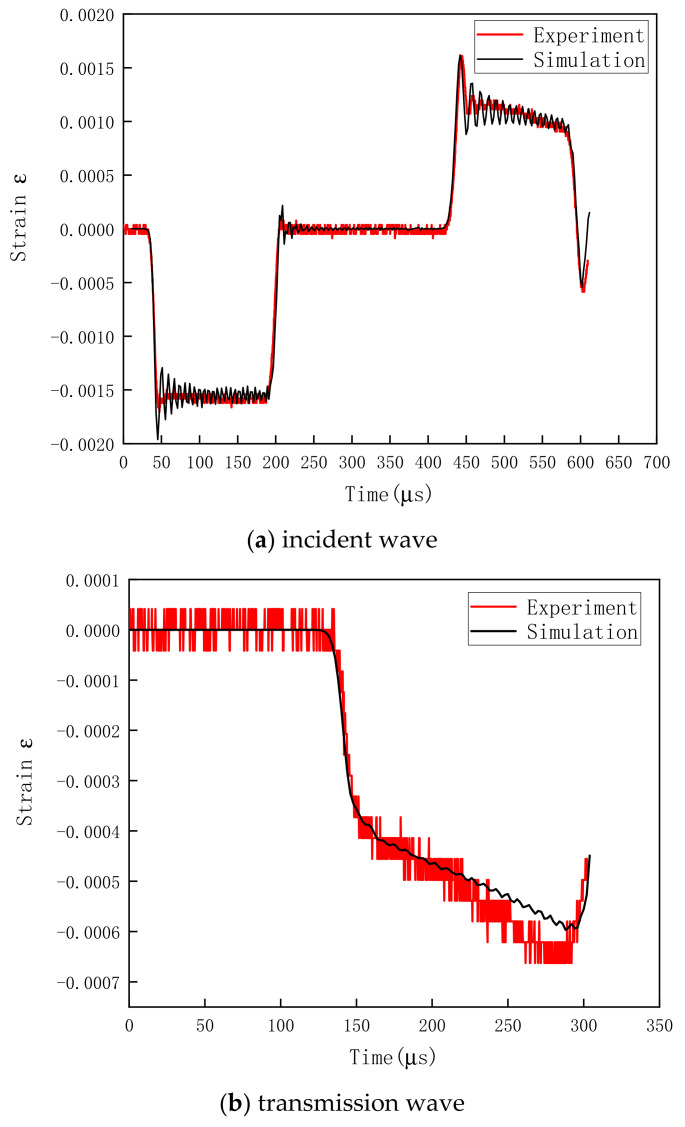
Comparison between strain waveforms generated during test and simulation.

Figure 15 compares the test and simulation results at the bullet velocity of 15.5 m/s. As shown, the strain–time curves of incident and transmission waves obtained in the numerical simulation matched well with the test result. This indicates that the dynamic compression process of HDPE could be accurately simulated by substituting the constitutive equation parameters determined by experimental fitting into LS-DYNA software.

## 5. Conclusions

To facilitate the use of PE materials in the design and research of insensitive ammunition, the dynamic and static compressive mechanical properties of HDPE and UHMWPE were comparatively investigated. The stress–strain curves of both materials at temperatures of −40–120 °C and strain rates of 0.001–5500 s^−1^ were obtained. The research findings suggest that both HDPE and UHMWPE showed strong viscoelastic plasticity, temperature-softening effect, and strain rate-strengthening effect. The HDPE exhibited better compression resistance and high-temperature resistance than the UHMWPE under the same working conditions. Moreover, the HDPE and UHMWPE had similar microscopic morphologies, both containing few branched chains. The calculation results showed that HDPE had a higher crystallinity than UHMWPE. Furthermore, a constitutive model for the two materials was built by fitting their yield stresses at different temperatures and strain rates. The results were validated by experimental results, indicating that the yield stress of PE materials at different temperatures and strain rates could be well predicted by the proposed constitutive model. Moreover, the constitutive model parameters obtained were substituted into LS-DYNA for simulation. The test result matched well with the simulation result of the HDPE dynamic compressive mechanical properties under the same working conditions. However, further research is needed, since the simulation of the temperature effect requires the secondary development of the material model in LS-DYNA.

## Figures and Tables

**Table 1:** Material properties.

	ρ (g/cm^3^)	*E* (MPa)	ν	Molecular Weight (g/mol)	$\mathrm{Melting}\;\mathrm{Enthalpy}\;\mathrm{of}\;100\%\;\mathrm{Crystallinity}\;\Delta H_{m}\;(J/g)$
HDPE	0.95	388	0.46	0.04–0.7 million	270.03
UHMWPE	0.98	597.28	0.46	6 million	288.42

**Table 2:** Parameters of the split Hopkinson pressure bar [8].

*E*_0_ (GPa)	Elastic Wave Velocity *C*_0_ (m/s)	Bar Diameter (mm)	Striker Length (mm)	Incident Bar Length (mm)	Transmission Bar Length (mm)
70	4991	14.5	400	1500	2000

**Table 3:** Compressive properties of HDPE and UHMWPE.

Strain Rate (s^−1^)	Temperature (°C)	Yield Stress of HDPE (MPa)	Yield Stress of UHMWPE (MPa)
0.1	25	28.03±0.15	21.60±0.18
0.01	25	23.78±0.11	18.60±0.20
0.001	25	20.35±0.09	14.64±0.09
0.001	50	13.31±0.10	10.65±0.17
0.001	70	8.17±0.09	6.69±0.14
0.001	90	6.18±0.03	3.49±0.12
0.001	120	2.18±0.05	1.49±0.08
0.001	−20	36.98±0.24	33.79±0.39
0.001	−40	41.55±0.15	39.03±0.33

**Table 4:** Parameters of the constitutive model.

	$\dot{\varepsilon }$	$\sigma_{0}\;(\mathrm{MPa})$	C	P	F	G
HDPE	$\left[0.001s^{-1},0.1s^{-1}\right]$	20.35	374.44	5.58	−3.98288	2.44658
	$\left[935s^{-1},5450s^{-1}\right]$				−1.36692	0.67368
UHMWPE	$\left[0.001s^{-1},0.1s^{-1}\right]$	14.64	1038.84	6.35	−5.18376	7.0655
	$\left[1300s^{-1},5500s^{-1}\right]$				−2.25395	2.18186

**Table 5:** Material model of split Hopkinson pressure bar.

MAT_PLASTIC_KINEMATIC		
ρ (g/cm^3^)	$\mathrm{Yield}\;\mathrm{Stress}\;\sigma_{s}\;(\mathrm{MPa})$	Poisson’s Ratio ν
2810	370	0.33

## Data Availability

The experimental and numerical modeling results are available upon request.
